# Beyond Vaccination: Persistent Meningococcal Risk in Anti-C5–Treated aHUS—Case Report and Review of Literature

**DOI:** 10.3390/jcm15083048

**Published:** 2026-04-16

**Authors:** Simona Matarese, Giacomo Brisca, Andrea Moscatelli, Marta Romanengo, Alessio Mesini, Marcello Mariani, Gabriele Mortari, Elio Castagnola, Micaela Gentile, Enrico Verrina, Gianluigi Ardissino, Edoardo La Porta

**Affiliations:** 1 Pediatric Emergency Room and Emergency Medicine Unit, Emergency Department, IRCCS Istituto Giannina Gaslini, 16147 Genoa, Italy; simonamatarese@gaslini.org; 2 Neonatal and Pediatric Intensive Care Unit and Intermediate Care Unit, IRCCS Istituto Giannina Gaslini, Via Gerolamo Gaslini 5, 16147 Genoa, Italy; giacomobrisca@gaslini.org (G.B.); andreamoscatelli@gaslini.org (A.M.); martaromanengo@gaslini.org (M.R.); 3 Pediatric Infectious Diseases Unit, Department of Pediatrics, IRCCS Istituto Giannina Gaslini, 16147 Genoa, Italy; alessiomesini@gaslini.org (A.M.); marcellomariani@gaslini.org (M.M.); eliocastagnola@gaslini.org (E.C.); 4 UOC Nephrology, Dialysis and Transplantation, IRCCS Istituto Giannina Gaslini, 16147 Genoa, Italy; gabrielemortari@gaslini.org (G.M.); enricoverrina@gaslini.org (E.V.); 5 UOC Nephrology, Guglielmo da Saliceto Hospital, AUSL Piacenza, 29121 Piacenza, Italy; micaela1989@gmail.com; 6 Center for HUS Prevention Control and Management, Pediatric Nephrology Dialysis, Transplant Unit Fondazione, IRCCS Ca’ Granda-Ospedale Maggiore Policlinico, 20122 Milano, Italy; ardissino@centroseu.org

**Keywords:** aHUS, C5 inhibitor, meningitis

## Abstract

**Background/Objectives**: Atypical hemolytic uremic syndrome (aHUS) is a rare, life-threatening thrombotic microangiopathy caused by dysregulation of the alternative complement pathway, often related to genetic mutations or autoantibodies. The introduction of complement C5 inhibitors, such as eculizumab and ravulizumab, has significantly improved renal and overall outcomes. However, complement inhibition impairs host defense against encapsulated bacteria, markedly increasing the risk of invasive infections, particularly *Neisseria meningitidis*. Vaccination against meningococcal groups ACWY and B, along with temporary antibiotic prophylaxis, is therefore recommended before initiating anti-C5 therapy. **Methods**: We report the clinical course of a 13-year-old boy with aHUS secondary to anti–complement factor H (CFH) autoantibodies and CFHR3–CFHR1 homozygous deletion, treated with C5 inhibitors. **Results**: Despite complete meningococcal vaccination and a previous course of antibiotic prophylaxis, the patient developed meningitis during ongoing complement inhibitor therapy. **Conclusions**: This case highlights that breakthrough invasive infections may occur despite adherence to recommended preventive strategies. It underscores the need for sustained clinical vigilance, timely vaccine boosters, and careful reassessment of the risk–benefit balance of continued complement inhibition therapy.

## 1. Introduction

Atypical hemolytic uremic syndrome (aHUS) is a thrombotic microangiopathy characterized by platelet consumption, mechanical, non-immune-mediated hemolysis, kidney and multiorgan damage due to endothelial injury mostly caused by the overactivation of the alternative complement pathway [1].

In the pediatric age, more than 80% of cases of aHUS are associated with complement abnormalities in genes encoding for regulatory proteins of the alternative pathway function (CFH or related genes, CFI, CFB, C3, CD46) or with the production of auto-antibodies against CFH of either IgG or IgM type [2].

In the past, the disease was typically burdened by a very severe prognosis, with the majority of cases progressing to end-stage kidney disease within months or a few years. Since 2009, the availability of a C5 inhibitor (Eculizumab) has dramatically improved the outcome of atypical HUS [3].

The drug is a humanized monoclonal antibody that binds to the complement component 5, preventing its cleavage into C5a and C5b, thus inhibiting the generation of the terminal membrane attack complex (MAC). Today, several other drugs modulating the complement function are available or in the registration process or in the course of trials for their use in several complement-mediated diseases, including aHUS (e.g., C3 inhibitor Pegcetacoplan or selective factor B inhibitor Iptacopan or selective factor D Danicopan) [4].

The complement system is part of the innate immune system, and is particularly important in the response against encapsulated bacteria and in several autoimmune conditions. Therefore, the inhibition of the complement system may expose patients to severe infections, including *Neisseria meningitidis* (*N. Meningitidis*) [5].

The risk of meningococcal infection in patients on C5 inhibition has been estimated from 550 to 2000-fold higher than that of the normal population based on several studies [6,7]. This risk makes vaccination mandatory in patients undergoing complement inhibition treatment; specifically, the Advisory Committee on Immunization Practices recommends both conjugated vaccines to ACWY and to B for patients on C5 inhibitor with booster doses according to the vaccination schedule [8] (Table 1). In case vaccination is not possible due to the rapidly progressive thrombotic microangiopathy (TMA), antibiotic prophylaxis against encapsulated bacteria should be started up to two weeks after vaccination.

Nevertheless, a risk for invasive infection remains regardless of vaccination. Thus, clinicians should maintain a high index of suspicion in front of specific symptoms in this group of patients and plan the safe discontinuation of C5 inhibitors in selected groups of patients. Herein, we describe a case of a thirteen-year-old boy who developed a *N. meningitidis* group B meningeal infection while receiving ravulizumab therapy despite full meningococcal vaccination and prior antibiotic prophylaxis.

## 2. Case Report

### 2.1. Patient History

A thirteen-year boy was followed since March 2017 in the nephrology department of the Giannina Gaslini Children’s Hospital in Genoa, Italy, resulting from the onset of atypical hemolytic uremic syndrome (aHUS) due to anti-complement factor H (CFH) antibodies of the IgG type with homozygous deletion of the CFHR3-CFHR1 gene. At onset, plasma exchange (PEX) alone was used to induce remission. In September 2017, when he was 6 years old, the patient experienced his first aHUS relapse and was successfully managed with the combination of eculizumab together with PEX and MMF at 1 g/m^2^/day for 6 months. Following the induction phase of C5i treatment, the patient was recommended to a progressive interval extension based on a target CH50 of <30%. Thus, from April 2020, the patient regularly received eculizumab (initially 900 mg and further adjusted to the weight gain with growth) every 4 weeks. Attempts to extend the dosing interval to 5 weeks were consistently followed by increases in the hemolysis index, proteinuria and hematuria, suggesting early TMA reactivation and precluding safe discontinuation of C5 inhibition. Anti-CFH antibody titers remained elevated over time (>100 AU/mL at onset [n.v. < 5.3] and 91 AU/mL two years later), further supporting the decision to maintain treatment. Therefore, in September 2023, the patient was switched to ravulizumab to ensure sustained complement blockade with a longer interval by administering the weight-based loading dose with regular further maintenance dose administrations every 8 weeks.

### 2.2. Vaccination and Prophylaxis

After anti-C5 therapy had been initiated, the patient had received a full course of meningococcal vaccination (A, C, W135, Y and B) together with antibiotic prophylaxis with amoxicillin for two weeks after vaccination, according to current recommendations and subsequently discontinued. A second dose of the anti-meningococcal type B vaccine was given in October 2017 and in January 2018. A booster dose of anti-B vaccination was given in January 2021.

### 2.3. Acute Infection Episode

In March 2024, exactly 8 weeks after the last ravulizumab infusion and 3 years and two months after the last booster dose, the patient presented at the emergency department with a fever (>39.5 °C), severe headache with nuchal rigidity and a positive Kernig sign and petechiae on the lower limbs and trunk.

Blood tests showed neutrophilic leukocytosis and elevated inflammatory markers. Coagulation factors were normal, and there was no evidence of hemolysis. With a strong suspicion of meningitis, in the absence of clinical signs of endocranial hypertension, a lumbar puncture was performed, and antibiotic therapy with ceftriaxone was promptly started.

**Figure 1:**
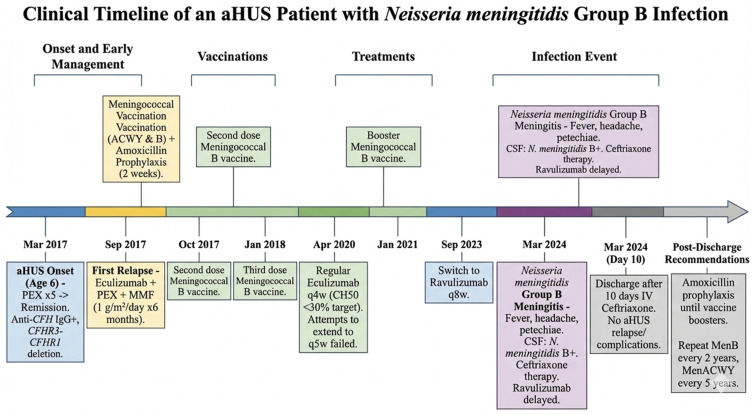
Clinical timeline of aHUS patient.

Cerebrospinal fluid analysis (CSF) showed increased cellularity (2164/mmc, mostly polymorphonuclear leucocyte), hyperproteinorrachia (89 mg/dL) and hypoglycorrhachia (24 mg/dL with contextual blood capillary glucose 102 mg/dL). The CSF culture resulted in a positive for the *N. meningitidis* group B. A brain MRI revealed no pathological findings. The administration of ravulizumab was temporarily withheld and postponed for two weeks. The patient was discharged after 10 days of intravenous antibiotic therapy with ceftriaxone, with the indication of repeating meningococcal B vaccination every 2 years and ACWY quadrivalent vaccination every 5 years. Amoxicillin prophylaxis was maintained until a booster dose of both vaccines had been given. He had no complications nor HUS relapse during hospitalization (Figure 1).

## 3. Revision of Literature and Discussion

The use of complement inhibitors is one of the major advances in the last two decades for treating TMA. Complement manipulation through inhibitors prevents endothelial damage and the activation of coagulation, blocking hemolysis and the formation of microthrombi; however, it increases the risk of infection from encapsulated bacteria. Although most epidemiological and mechanistic data derive from studies on eculizumab, largely due to its longer clinical use, ravulizumab shares the same molecular target and, ultimately, exerts its effect through sustained C5 inhibition and a membrane attack complex (MAC) blockade. Nevertheless, potential differences in susceptibility to invasive infections caused by encapsulated organisms between the two agents cannot be definitively excluded. Data specifically regarding meningococcal infections during ravulizumab therapy are extremely limited. To date, only one case has been reported in the literature [9]. Therefore, current knowledge on infectious risk under ravulizumab largely derives from experience with eculizumab.

Meningococcal vaccine preparations include a quadrivalent polysaccharide A, C, W-135, Y meningococcal vaccine or conjugated vaccines to A, C, W-135, Y (men-ACWY) and conjugated vaccine to B (Men-B). Although the quadrivalent polysaccharide and conjugated vaccines protect against the same serogroups, major differences in immunogenicity have been noted between these preparations: conjugation seems to confer a longer protection through the activation of T cells [10].

Several cases of meningococcal infection have been reported in patients treated with eculizumab despite prior vaccination.

In 2013, Struijk et al. [11] described a patient treated with eculizumab and immunosuppressive drugs after renal transplantation who developed meningococcal sepsis despite regular vaccination for the same serotype. Although the less immunogenic, non-conjugated polysaccharide vaccine was used. In addition, vaccination was performed while the patient was already on immunosuppressive treatment.

A further case of meningococcal bacteremia by *N. meningitidis* W135 after 30 months from vaccination has been described in a child treated with eculizumab for aHUS, despite antibiotic prophylaxis with amoxicillin. After the acute phase, vaccine responses were tested, showing suboptimal titers and intermediate penicillin sensitivity [12]. Therefore, the duration of protection of the vaccine remains uncertain, especially for MEN-B vaccines [13]. A clinical trial that assessed antibody persistence in adolescents (11–17 years of age) who received two doses of 4CMen-B showed that only 64% of them had protective titers after 18–24 months [14]. Moreover, several works have shown that non-groupable strains of *N. meningitidis* can cause invasive meningococcal disease in patients receiving eculizumab [6]. In February 2017, a survey promoted by the Centers for Disease Control and Prevention in the US identified 16 cases of meningococcal disease in eculizumab recipients in 8 years. The majority of these cases were due to non-groupable *N. meningitidis* and involved patients who had undergone vaccination (at least one dose of meningococcal vaccine before the disease occurred). These pathogens are rarely responsible for invasive infection in the general population.

Interestingly, several cases of *Neisseria gonorrhoeae* invasive infection in patients treated with eculizumab are reported in the literature, suggesting that bacterial dissemination is more likely in patients on C5 inhibition [15].

Although durable protection is particularly important in individuals receiving anti-C5 therapy, especially if prescribed lifelong or in combination with other immunosuppressant dampening humoral responses, vaccination schedules may vary from country to country in the absence of shared international guidelines, and clear recommendations regarding booster timing are still lacking.

Given that many patients remain on complement inhibitors for years, greater attention should be paid to booster administration and to periodic reassessment of vaccine-induced immunity. This is particularly relevant in light of reports showing waning antibody titers over time, especially for Men-B vaccines, and considering that long-term complement blockade may further increase vulnerability to invasive diseases.

Notably, in several of the reported cases of meningococcal infection occurring during eculizumab therapy, the causative strains were either non-groupable or belonged to serogroups not fully covered by available vaccines. Since strain typing was not performed, we cannot determine whether the infecting *N. meningitidis* strain expressed antigens targeted by the Men-B vaccine. Therefore, infection with a non-vaccine-covered strain cannot be excluded, and the present case cannot be interpreted as definitive vaccine failure. In other cases, patients had received less immunogenic formulations, such as unconjugated polysaccharide vaccines, or had been vaccinated under conditions of concomitant immunosuppression, potentially limiting the development of robust and durable protective responses. These observations further support the need for optimized vaccination strategies, preference for conjugated vaccines when available, and careful planning of booster schedules in this high-risk population.

In addition, the duration of protection following Men-B vaccination remains uncertain, particularly in patients receiving long-term complement inhibition. Although serological response can be assessed through rabbit serum bactericidal antibody (rSBA) assays—where titers ≥ 1:8 are considered protective in immunocompetent individuals—the clinical relevance of such measurements in C5-inhibited patients is debated. Terminal complement blockade impairs the membrane attack complex (MAC) formation and reduces both bactericidal activity and opsonophagocytosis, even in the presence of detectable specific IgG and C3 opsonization. Therefore, apparently protective in vitro titers may not translate into effective in vivo protection [16,17].

For this reason, several authorities advocate long-term antibiotic prophylaxis [18]. However, prolonged broad-spectrum prophylaxis raises concerns regarding antimicrobial resistance and microbiome disruption. A pragmatic and individualized approach may, therefore, be appropriate. In highly adherent patients with immediate access to emergency medical care, strict clinical vigilance and urgent evaluation at the first sign of fever may represent a stewardship-conscious strategy [6,19]. When prophylaxis is deemed necessary, narrow-spectrum agents, such as oral penicillin V, should be preferred.

Despite that, immunity against meningococcal disease could also be achieved through bacterial destruction via the opsonophagocytic method, which does not rely on a fully functional terminal complement pathway. It has been demonstrated that vaccination confers protection against meningococcal disease in patients with inherited complement deficiencies, while in patients treated with eculizumab, the inhibition of C5 cleavage does not allow the release of the pro-inflammatory peptide C5a, impeding the opsonophagocytic activity against encapsulated meningococcal strains of serogroup B or C, even in immunized patients [16] with detectable IgG levels and C3 opsonization [20].

For these reasons, some healthcare providers and public health organizations in the UK and France propose penicillin as prophylaxis during eculizumab treatment (or macrolides for patients with penicillin allergies). Long-term penicillin prophylaxis is generally safe, although meningococcal disease has been reported in eculizumab patients receiving penicillin chemoprophylaxis caused by strains with penicillin resistance or intermediate sensitivity [21].

Due to the increasing prevalence of meningococcal strains with penicillin resistance prophylaxis, the effectiveness of preventing meningococcal disease has not been definitively established, and its use in clinical practice is still controversial. Finally, for many new complement inhibitors, such as the C3 inhibitor pegcetacoplan, the risk for invasive infections by encapsulated bacteria is still not known, but it is probably higher than the C5 inhibitors because the alternative pathway is blocked upstream.

The high infectious risk and the impossibility of fully preventing infections despite full vaccination or antibiotic prophylaxis pushed clinicians to look at assessing the correct timing for discontinuing therapy, thereby reducing the risk of infection. Ardissino et al., in 2014 [2], showed that discontinuation of eculizumab is generally safe in atypical SEU with stable remission and the dysregulation of complement-related diseases. While the risk of relapse in genetically negative forms of idiopathic HUS is negligible compared to primary HUS (genetic mutations without trigger), with a relapse rate of 18.7% and 66.7%, respectively.

However, discontinuation is not suggested for patients with anti-CFH antibodies because of the higher risk of relapse [22]. Other studies focused on the type of genetic mutation, identifying CFH variants as the ones with the highest risk of relapse [23]. Finally, clinical features must be taken into account before scheduling treatment discontinuation, such as kidney function, disease severity at presentation and extra-renal manifestations. Caregivers should be counselled about the risk of relapse, particularly during acute infections, which are known triggers of complement activation. In clinical practice, infectious episodes in these high-risk patients usually require hospital evaluation, allowing prompt laboratory monitoring and the early detection of TMA reactivation.

In our case, we attempted to discontinue the C5 blockade. However, early laboratory signs of TMA reactivation did not allow complete withdrawal of therapy. Therefore, the patient was transitioned from a 4-week eculizumab regimen to an 8-week ravulizumab schedule. Further discontinuation attempts may be considered in the future after careful safety evaluation.

In conclusion, the risk of meningococcal infection in patients on C5i therapy remains high even in vaccinated patients. For newer complement inhibitors, such as ravulizumab, which shares the same mechanism of action as eculizumab through C5 blockade, data regarding long-term infectious susceptibility remain limited. Although both agents inhibit terminal complement activation, differences in pharmacokinetics, dosing intervals and duration of complement suppression may theoretically influence infectious risk. To date, the literature on meningococcal disease has been largely derived from experience with eculizumab, and whether the risk profile fully overlaps with that of ravulizumab remains to be clearly established. Dedicated post-marketing surveillance and real-world data will be essential to better define the infectious risk associated with prolonged C5 inhibition with different agents.

## Figures and Tables

**Table 1:** Vaccination schedule for patients on C5 inhibitors [8].

	First Dose	Second Dose	Booster Doses
Men-ACWY	At least 2 weeks before starting therapy with C5i	8 weeks after the first dose	Every 5 years if treatment with C5i continues
Men-B	At least 2 weeks before starting therapy with C5i	4 weeks after the first dose	1 year after completing the initial series, then every 2–3 years if treatment with C5i continues

## Data Availability

No new data were created or analyzed in this study. Data sharing is not applicable to this article.
